# Encephalomyocarditis virus (EMCV): An overlooked threat to primates

**DOI:** 10.1371/journal.pntd.0014409

**Published:** 2026-06-23

**Authors:** Inestin Amona, Charles Kumakamba, Clément Labarrere, Bernard Davoust, Florence Fenollar, Oleg Mediannikov

**Affiliations:** 1 IHU Méditerranée Infection, CEDEX 05, Marseille, France; 2 Risques Infectieux Tropicaux Microorganismes Emergents (RITMES), Aix-Marseille University, AP-HM, SSA, Marseille, France; 3 Microbes Evolution Phylogénie et Infection (MEPHI), Aix-Marseille University, AP-HM, Marseille, France; 4 Institut de Recherche pour le Développement (IRD), Marseille, France; Ross University School of Veterinary Medicine, SAINT KITTS AND NEVIS

## Abstract

Many emerging zoonotic viruses pose major risks to animal and human health, with most recent epidemics of viral origin. This review focuses on encephalomyocarditis virus (EMCV), a member of the *Picornaviridae* family and the *Cardiovirus* genus with significant but largely underrecognized zoonotic potential. While EMCV has not yet caused major public health crises, its broad host range and rodent reservoir suggest a wider ecological impact. It is typically detected only during high-fatality outbreaks, particularly in domestic animals and non-human primates (NHPs), which are among the most vulnerable hosts. Multiple fatal outbreaks in captive and semi-captive NHPs have been documented. Human infections appear rare, yet the prevalence of antibodies suggests widespread exposure. EMCV remains largely absent from diagnostic panels and its epidemiology is poorly understood. We argue that EMCV warrants much closer attention due to its ability to cause severe disease in NHPs and its potential risk to humans. This review synthesizes current knowledge on the biology, epidemiology, pathogenicity, diagnosis, and prevention of EMCV in primates.

## 1. Introduction

Encephalomyocarditis virus (EMCV) is a Picornavirus, widely distributed worldwide, and responsible for infections in a broad range of domestic and wild animal hosts, as well as in humans [1]. Pigs are considered the most frequently and severely affected species, with infection characterized by severe acute myocarditis, encephalitis, and sudden death in piglets, as well as reproductive failure in sows [2–4]. In addition to pigs, some wild animal species, such as non-human primates (NHPs) and elephants, are highly susceptible and often severely affected [5–8]. EMCV was first described in 1944–1945 following fatal encephalomyocarditis in captive NHPs, a gibbon (*Hylobates* sp.) and a chimpanzee (*Pan troglodytes*) housed at the Anthropoid Ape Research Foundation in Dania Beach, Florida, USA [9]. In 1946, Mengo virus, later recognized as a strain of EMCV, was isolated from the cerebrospinal fluid of a paralyzed captive rhesus macaque (*Macaca mulatta*), as well as from mosquitoes and a mongoose, at the Mengo Research Institute in Entebbe, Uganda [10]. The Mengo virus was subsequently identified from a laboratory worker at the same institute who presented with a febrile illness accompanied by encephalitis, ~5 months after its initial detection in the rhesus macaque [11].

**Fig 1:**
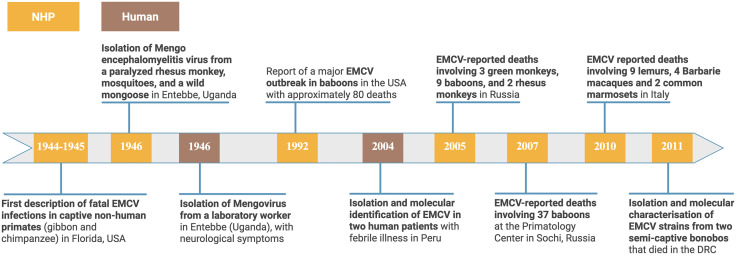
Timeline summarizing key events related to the detection, isolation, and reported cases of encephalomyocarditis virus (EMCV) infection in human and nonhuman primates (NHPs). Created in BioRender. FENOLLAR, F. (2026) https://BioRender.com/cs8mssl.

Since then, fatal infections, along with successful virus isolation, have been increasingly reported in various species of captive and semi-captive NHPs in zoological parks, primatology research centers, and sanctuaries worldwide (Fig 1) [6,12–15].

Documented cases in NHPs include chimpanzees [6,9], siamangs [7], golden lion tamarins [7], black-and-white colobus monkeys [7], gibbons [9], squirrel monkeys [13], mandrills [13], bonobos [14], rhesus macaques [10,15], orangutans [16], grivets [17], lemurs [18], common marmosets [18], baboons [18], and barbary macaques [18,19]. Rodents (rats and mice) are considered the natural reservoirs of the virus, transmitting it to other animal species primarily via the fecal-oral route [20,21].

Although infection with EMCV and severe cases of the disease are frequently reported in animals, this virus is recognized as a potential zoonotic agent [1,11]. Accordingly, enhanced surveillance and comprehensive investigations are necessary, given its significance in animal health and its broad host range. Human cases reported to date, identified through serological assays or blood sample analyses, remain limited in number and have predominantly been associated with either asymptomatic infections [22–25] or nonspecific clinical manifestations, including febrile syndromes [11,26].

Despite the low incidence of documented acute illness in humans, several reports suggest that human exposure to EMCV may be relatively common. Serological surveys have demonstrated EMCV seroprevalence in human populations across multiple countries, including Australia [22], Peru [23], Mexico [24], China [25], Hawaii [27], and Austria [28,29].

Furthermore, a study conducted in Peru by Oberste and colleagues demonstrated the presence of EMCV RNA in the serum of two human patients with febrile illness, followed by successful virus isolation in Vero cells [26]. EMCV was also isolated from the blood and serum of a human case presenting neurological symptoms in Uganda, using laboratory mice [11]. Although there is currently no direct evidence of EMCV transmission from animals to humans, some studies have established an association between human EMCV infections and exposure to animals [11,23,28–30].

Despite the known susceptibility of NHPs to EMCV infection, available epidemiological data for both humans and primates remain limited. Research on EMCV infection is scarce and often outdated.

While severe cases associated with EMCV have been documented in captive and semi-captive NHPs, the potential for undetected severe human cases or silent circulation of the virus within wild NHPs populations remains hypothetical. Furthermore, although great apes and humans share close phylogenetic relationships and may exhibit susceptibility to similar pathogens, such as Ebola virus, their clinical manifestations differ markedly. To date, the existence of severe forms of EMCV disease in humans has not been conclusively demonstrated, and no human fatalities associated with the virus have been reported [31,32].

Therefore, it is crucial to monitor and gather relevant data on zoonotic pathogens in both humans and NHPs in order to anticipate and prevent potential threats to public health and wildlife conservation.

This review provides a synthesized overview of EMCV research in primates and underscores the limited epidemiological data available for humans and NHPs.

## 2. Methods

### 2.1. Literature search

A literature review was conducted to identify all publications reporting cases of EMCV infection in humans and NHPs. Searches were performed in the PubMed and Google Scholar databases, as well as using the Google search engine. Predefined keywords related to EMCV and NHPs or humans were combined using the Boolean operators “AND” and “OR” to generate multiple search combinations. No restrictions on language or publication date were applied, allowing for the inclusion of both historical and recent studies.

### 2.2. Study selection

All identified references were initially reviewed based on their titles and abstracts. Articles considered relevant to the objectives of the review were subsequently retrieved and analyzed in full text. Eligible publications included case reports, outbreak investigations, and epidemiological studies documenting EMCV infection in NHPs or humans, as well as relevant data reported in other animal species.

Given the limited amount of available data and the historical nature of many studies, all publications reporting EMCV infection in humans and NHPs since the virus was first identified in 1944 were included. Study selection was based on relevance to the review objectives, rather than strict inclusion/exclusion criteria.

### 2.3. Data extraction and synthesis

Data extracted from the selected studies included the affected NHPs species, geographic location, year of publication, study type, and key findings related to EMCV infection. A narrative synthesis was then performed to summarize the available evidence of EMCV infection in humans and NHPs.

## 3. Classification and biological characteristics of EMCV

EMCV is a small, non-enveloped, single-stranded RNA virus belonging to the *Picornaviridae* family and the *Cardiovirus* genus [1,33].

**Fig 2:**
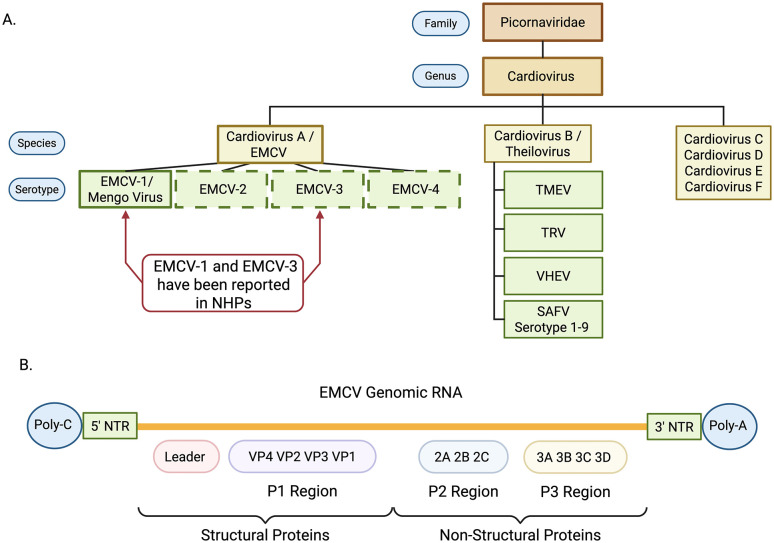
Classification (A) and Genomic organization (B) of encephalomyocarditis virus (EMCV). The encephalomyocarditis virus (EMCV) belongs to the *Picornaviridae* family and the *Cardiovirus* genus. This genus is currently classified into six species according to the International Committee on Taxonomy of Viruses (ICTV): *Cardiovirus A*, which includes four serotypes of EMCV; *Cardiovirus B*, comprising 14 types, including Theiler’s murine encephalomyelitis virus (TMEV), Thera virus (TRV), Vilyuisk human encephalomyelitis virus (VHEV), and Saffold virus (SAFV); as well as *Cardiovirus C* (3 types), *Cardiovirus D* (11 types), *Cardiovirus E* (1 type), and *Cardiovirus F* (1 type). In the diagram, solid lines and borders represent well-established taxonomic classifications and verified serotypes, whereas dashed lines and borders indicate species or serotypes whose classification is currently under debate within the scientific community. Created in BioRender. FENOLLAR, F. (2026) https://BioRender.com/mkgj9xa*.*

The genus *Cardiovirus* is subdivided into several species (A to F), comprising 32 genetic types distinguished by means of phylogenetic analysis (*Cardiovirus* A: 2 types; *Cardiovirus* B: 14 types; *Cardiovirus* C: 3 types; *Cardiovirus* D: 11 types; *Cardiovirus* E: 1 type; *Cardiovirus* F: 1 type) [34–49]. EMCV belongs to the species *Cardiovirus* A and includes two serotypes recognized by the International Committee on Taxonomy of Viruses (ICTV): EMCV-1 (*Cardiovirus* A1) and EMCV-2 (*Cardiovirus* A2) (Fig 2A) [39,40].

### 3.1. Genetic diversity, host range, and geographic distribution

EMCV, a member of the *Picornaviridae* family, exhibits significant genetic diversity, initially described in the literature in terms of lineages, “serotypes”, and strains [41,42]. Among the serotypes, EMCV-1 is the most extensively characterized and the most widely distributed worldwide. It comprises eight lineages (A–H), which are further structured into four major evolutionary clades (I–IV) [41,42]. These clades encompass diverse strains of EMCV and Mengo virus, reported across a broad range of hosts, including several animal species [2,4,7–13,21], and arthropods [10], as well as humans [11,26]. This group includes historical strains such as EMCV Ruckert, isolated from a chimpanzee in Florida, United States [9], and Mengo M, isolated from a rhesus monkey in Uganda [10].

The recently described EMCV-2 serotype includes the highly divergent strain RD-1338, isolated from a captive wood mouse (*Apodemus sylvaticus*) in Germany [43], as well as other viral variants frequently detected in wild hazel dormice (*Muscardinus avellanarius*) in England [44].

In addition, a third putative serotype (EMCV-3), comprising the Sing-M100-02 and Sing-M105-02 strains isolated from captive orangutans in Singapore, has been proposed on the basis of marked phylogenetic divergence. However, this classification remains provisional, as comprehensive serological characterization has not yet been performed [16,40]. Similarly, divergent viruses identified in rats (*Bandicota indica*) in Vietnam have been suggested to represent a novel serotype (EMCV-4) [41]. Nevertheless, as with EMCV-3, this designation is currently based exclusively on genetic and phylogenetic evidence, lacking serological confirmation [40].

### 3.2. Genome organization and structure

The EMCV genome is approximately 7.8 kb in length and consists of a single large open reading frame (ORF) flanked by 5’ and 3’ untranslated regions (UTRs) and ending in a poly-A tail. The coding region comprises the leader protein (L), the P1 region encoding the structural proteins VP4, VP2, VP3, and VP1 (genes 1A–1D), and the P2 and P3 regions encoding the nonstructural proteins 2A–2C and 3A–3D, respectively [1]. A poly(C) segment of ~150 nucleotides is present in the 5’ UTR, although its length can vary depending on the viral strain (Fig 2B) [45,46].

## 4. Epidemiological characteristics of EMCV in NHPs

### 4.1. Reported epizooties in NHPs

**Fig 3:**
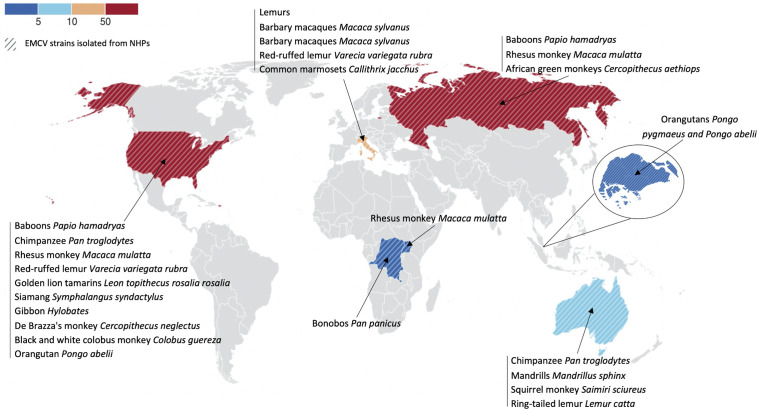
Global distribution of cumulative encephalomyocarditis virus cases reported in NHPs from 1944 to 2025. The colored areas (red, blue, orange, and cyan) indicate the geographical regions where EMCV cases have been reported in NHPs and the virus isolation. The most significant cases have been reported in the USA and Russia. Map created with Datawrapper; base map layer (administrative boundaries) provided by Natural Earth (https://www.naturalearthdata.com).

Since its first identification in 1944–1945 in the USA from a gibbon and a chimpanzee, fatal outbreaks and sporadic cases of EMCV-associated infections have been reported in various captive and semi-captive NHPs species. These cases have occurred in the USA [6,7], Africa [10], Australia [13], Asia [16], Russia [17], and Italy (Fig 3) [18,19].

In the USA, following the initial report of two sporadic cases involving the deaths of a gibbon on November 1944 and a chimpanze 6 weeks later at the Anthropoid Apes Research Foundation in Florida, the first confirmed epidemic mortality events associated with EMCV in NHPs occurred between 1977 and 1978.

These outbreaks resulted in the deaths of a De Brazza monkey (*Cercopithecus neglectus*) and three chimpanzees at the Lion Country Safari Zoo in West Palm Beach, Florida [6]. The cases were recorded on January 1977 (chimpanzee 1), December 1977 (De Brazza’s monkey), and February 1978 (chimpanzees 1 and 2). Between January and August 1985, another epidemic was recorded, resulting in the death of eight NHPs at the Audubon Park Zoo in New Orleans, Louisiana.

The affected animals included three black and white ruffed lemurs (*Varecia variegata*), one black-and-white colobus monkey (*Colobus guereza*), two golden lion tamarins (*Leontopithecus rosalia rosalia*), and two siamangs (*Symphalangus syndactylus*) [7].

In subsequent years, sporadic cases and several fatal epizootics were reported in various NHPs species. These included an orangutan (*Pongo abelii*) in January 1987 [12], baboons (*Papio hamadryas*) in 1992 [47], and rhesus macaques in October 2008 and December 2009 [15]. The baboon epizootic represents one of the most significant events, resulting in the death of approximately 80 individuals [47].

More recently, between March and April 2019, confirmed EMCV-associated mortality was reported in a zoo in central Florida (USA), affecting three mandrills (*Mandrillus sphinx*) and one lion-tailed macaque (*Macaca silenus*) [40].

In Asia, the first reported cases of EMCV infection in NHPs occurred between July 2001 and January 2002, when four orangutans (*Pongo pygmaeus* and *Pongo abelii*) died at the Singapore Zoological Gardens [16].

In Australia, fatal EMCV infections were reported at Taronga Zoo between March 1987 and June 1995, affecting a ring-tailed lemur (*Lemur catta*), a squirrel monkey(*Saimiri sciureus*), three mandrills, and a chimpanzee [13].

In Russia, the first documented cases date back to the 1970s, involving the deaths of two rhesus macaques, followed by multiple mortality events affecting baboons, green monkeys (*Cercopithecus aethiops*), and rhesus macaques, across the country [17,41,48]. For instance, between March and April 2007, 37 out of 46 semi-captive hamadryas baboons housed at the Primate Institute in Sochi died due to EMCV infection [41].

In Italy, two confirmed outbreaks of EMCV were recorded at the Natura Viva Zoo in northern Italy between October 2006 and February 2008. The first outbreak led to the deaths of 18 captive NHPs, including black lemur (*Eulemur macaco macaco*), ring-tailed lemurs, red-ruffed lemurs (*Varecia rubra*), white-fronted lemurs (*Eulemur albifrons*), common marmosets (*Callithrix jacchus*), and barbary macaques (*Macaca sylvanus*) [18].

The second outbreak, occurring between September and November 2008, resulted in the death of three red-ruffed lemurs [18]. Five years later, another outbreak-associated with a confirmed EMCV infection, occurring between September and November 2012, resulted in the deaths of four barbary macaques at the Rescue Center for Wild and Exotic Animals in central Italy [19].

In Africa, the first reported case of EMCV infection in NHPs was reported in 1946. It involving a paralyzed captive rhesus monkey at the Entebbe Research Institute in the Mengo district of Uganda, [10]. Subsequently, in 2003, two confirmed deaths associated with EMCV infection were recorded in semi-captive bonobos at the Lola ya Bonobo sanctuary in the Democratic Republic of Congo (Table 1) [14].

**Table 1:** Summary of deaths associated with EMCV in nonhumain primates species in different geographical regions of the world.

Date of death	NHPs species	Number of deaths	Geographic region	Reference
1944	Gibbon (*Hylobates* sp.)	1	USA	[9]
1944-1945	Chimpanzee (*Pan troglodytes*)	1	USA	[9]
1946	Rhesus macaque (*Macaca mulatta*)	1	Uganda	[10]
1947	Rhesus macaque	1	Uganda	[10]
1970	Rhesus macaques	2	Russia	[48]
1977	De Brazza’s monkey (*Cercopithecus neglectus*)	1	USA	[6]
1978	Chimpanzee (*Pan troglodytes*)	3	USA	[6]
1978-1979	Baboons (*Papio hamadryas*)	4	Russia	[48]
1982-1988	Rhesus macaques	6	Russia	[17]
1982-1988	Baboons (*Papio hamadryas*)	15	Russia	[17]
1985	Black and white ruffed lemur (*Varecia variegata*)	3	USA	[7]
1985	Black and white colobus monkey (*Colobus guereza*)	1	USA	[7]
1985	Golden lion tamarins (*Leontopithecus rosalia rosalia*)	2	USA	[7]
1985	Siamang (*Symphalangus syndactylus*)	2	USA	[7]
1987	Orangutan (*Pongo abelii*)	1	USA	[12]
1987	Squirrel monkey (*Saimiri sciureus*)	1	Australia	[13]
1987	Mandrills (*Mandrillus sphinx*)	2	Australia	[13]
1991	Mandrills (*Mandrillus sphinx*)	1	Australia	[13]
1992	Chimpanzee (*Pan troglodytes*)	1	Australia	[13]
1992	Baboons (*Papio hamadryas*)	80	USA	[47]
1995	Ring-tailed lemur (*Lemur catta*)	1	Australia	[13]
2001-2002	Orangutans (*Pongo pygmaeus and Pongo abelii*)	4	Singapore	[16]
2001-2003	Green monkeys *(Cercopithecus aethiops)*	3	Russia	[17]
2001-2003	Baboons (*Papio hamadryas*)	9	Russia	[17]
2001-2003	Rhesus macaques	2	Russia	[17]
2003	Bonobos (*Pan panicus*)	2	RDC	[14]
2006-2007	Lemurs (*Eulemur macaco macaco, Lemur catta, Varecia variegata, Varecia rubra, Eulemur albifrons*)	9	Italy	[18]
2006-2007	Barbary macaques (*Macaca sylvanus*)	4	Italy	[18]
2006-2007	Common marmosets (*Callithrix jacchus*)	2	Italy	[18]
2007	Baboons (*Papio hamadryas*)	37	Russia	[41]
2008	Red-ruffed lemur (*Varecia variegata rubra*)	3	Italy	[18]
2008-2009	Rhesus macaques	6	USA	[15]
2012	Barbary macaques (*Macaca sylvanus*)	4	Italy	[19]
2019	Mandrills (*Mandrillus sphinx*)	3	USA	[40]
2019	Lion-tailed macaque (*Macaca silenus*)	1	USA	[40]

### 4.2. Risk factors

EMCV infection in NHPs is strongly associated with environmental risk factors, particularly in captive and semi-captive settings. The primary driver of infection is exposure to rodents, notably those of the genus *Rattus*, which serve as the main reservoirs of the virus [25,26]. Transmission typically occurs indirectly through the contamination of food, water, or surfaces by rodent excreta. Numerous cases of sporadic infections or reported outbreaks have been associated with the proliferation of infected rodents in sites housing NHPs [6,13,14,18,19]. These observations are consistent with those reported in pigs, where farm-level infections have been frequently traced back to the presence of infected rodents [52,53]. Furthermore, captive conditions characterized by insufficient biosecurity, including inadequate rodent control, inappropriate food storage, and infrastructure facilitating rodent intrusion, significantly increase the risk of exposure [13]. Ecological factors, including seasonal variations that influence rodent population dynamics and the environmental stability of the virus (favored by its non-enveloped structure), may further modulate transmission risk [6]. However, the role of host-specific factors, such as differential susceptibility among NHP species and across age groups, remains poorly characterized due to a lack of comparative studies and the predominance of case reports.

### 4.3. Interspecies barrier crossing

The epidemiology of EMCV in captive and semi-captive settings highlights its broad host tropism, as evidenced by its ability to infect a wide range of mammalian hosts, including multiple NHP species simultaneously within the same outbreak.

However, rather than indicating frequent interspecies transmission or host-adaptive barrier crossing, these reported cases are more likely explained by a shared-source spillover. This occurs through exposure to a common environmental source, particularly in contexts where different species coexist or share contaminated food and water resources [6,7,13].

Several outbreaks illustrate this pattern of multi-species involvement, without evidence of direct transmission between hosts. At Taronga Zoo in Australia, EMCV infection was associated with the deaths of a diverse array of taxa, including a ring-tailed lemur, a squirrel monkey, mandrills, a chimpanzee, a pygmy hippopotamus, and Goodfellow’s tree kangaroos [13]. Similarly, at Audubon Park Zoo in New Orleans, fatalities affected several NHPs species alongside non-primate mammals such as a Thomson’s gazelle and a dromedary [7]. Comparable multi-species outbreaks have been reported at Lion Country Safari Zoo in Florida and at a wildlife rescue center in Italy, involving both primates and non-primate species [6,19]. The recurrence of such diverse multi-species events underscores the virus’s lack of host specificity. Conceptually, these dynamics further support the hypothesis that transmission is primarily driven by environmental contamination.

Current evidence consistently identifies rodents as the principal reservoir and source of infection, with transmission occurring through the ingestion of food or water contaminated by rodent excreta or secretions, or through contact with infected carcasses [7,12,18,42]. This model provides a parsimonious explanation for the simultaneous infection of multiple species. Furthermore, the frequently reported seasonal clustering during colder months warrants further interpretation [6,13,15,17]. This seasonal pattern may reflect ecological changes in rodent populations, including increased shelter-seeking behavior in human-managed environments, as well as enhanced environmental persistence of the nonenvelopped virus at lower temperatures. Such factors synergistically increase the likelihood of environmental contamination and cross-species exposure among susceptible hosts.

### 4.4. Natural reservoirs

Rodents, particularly members of the *Muridae* family, are widely considered key hosts in the ecology of EMCV and are acting as primary sources of environmental contamination through viral shedding in feces and urine [20,21,49].

Epidemiological investigations have consistently reported associations between EMCV outbreaks in NHPs, domestic animals, and the presence of rodents in shared environments such as zoos, sanctuaries, primate centers, and breeding facilities, particularly in proximity to food storage areas and water sources [15,42,47,50,51]. For instance, an epizootic in pigs in central-western New South Wales was linked to a mouse (*Mus musculus*) infestation, resulting in 47 confirmed outbreaks across 37 farms and over a thousand pig deaths [52].

Molecular and serological evidence further supports this association. EMCV RNA and specific antibodies have been detected in both rodents and affected animal species [10,16,51,54]. In several settings, viral strains isolated from rodents have shown a high degree of genetic similarity to those identified in outbreak-associated species. For example, EMCV strains detected in rodents were nearly identical (98–99.9% nucleotide identity) to those associated with elephant mortalities in zoos in South Africa and France [42,55], and similar findings have been reported in pig farms [51]. In one U.S. zoo, EMCV was detected in multiple rats and mice collected from areas where NHP deaths and seropositive animals had been identified [7].

Together, these findings support a common-source exposure model in which rodents act as potential reservoir hosts and sources of spillover, with transmission to NHPs and other species most likely occurring through indirect exposure to contaminated food, water, or environments, rather than through direct contact. However, the precise origin of infection in individual outbreaks often remains difficult to conclusively determine.

Crucially, a distinction must be made between experimental competence and natural maintenance. Despite strong epidemiological and molecular links, evidence for stable, long-term maintenance of EMCV in wild rodent populations under natural conditions remains limited. Much of the current understanding of rodent competence derives from experimental studies. Under controlled conditions, rats have been shown to develop subclinical infections, shed virus for extended periods, and transmit infection horizontally to conspecifics [20,57]. Viral persistence has also been demonstrated in lymphoid tissues for several weeks post-infection [20], supporting the potential for prolonged carriage.

In contrast, field-based longitudinal data documenting sustained viral circulation within natural rodent populations are scarce. This distinction is important, as experimental findings may not fully reflect the complex ecological dynamics in the wild. Additionally, susceptibility appears to vary among rodent species: while rats (*Rattus* spp.) often exhibit subclinical carriage with prolonged shedding, the high mortality seen in some mice species might limit their role as long-term reservoirs [17].

**Fig 4:**
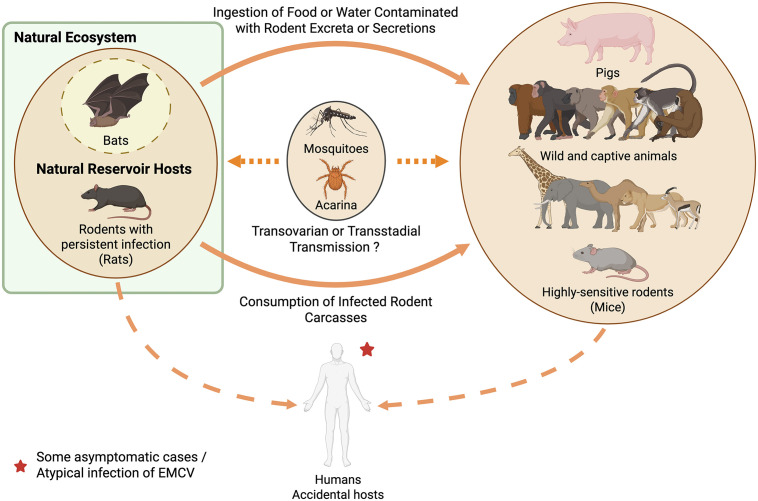
Reservoir hosts and transmission cycle of encephalomyocarditis virus. This diagram illustrates the multi-host ecology of EMCV, where rodents serve as the primary natural reservoirs maintaining the virus within the natural ecosystem through persistent infections, with bats also potentially involved in this maintenance. Transmission to other species occurs via two main reported pathways: the ingestion of food or water contaminated with rodent excreta or secretions, and the consumption of infected rodent carcasses. The potential role of arthropod vectors (mosquitoes and Acarina) in transovarian or transstadial transmission remains a hypothesis requiring further investigation. A wide range of susceptible hosts can be infected, including domestic pigs, various wild and captive animals (such as primates and elephants), and highly sensitive rodents like mice, while humans are considered accidental hosts. In the diagram, solid lines represent reported and verified transmission routes, while dashed lines indicate plausible hypotheses. Created in BioRender. FENOLLAR, F. (2026) https://BioRender.com/cxhowt9.

EMCV has been successfully isolated from a range of wild rodent species, including mice (*Mus* spp.), rats, *Mastomys* spp., dormice (*Myoxus glis*), cotton rats (*Sigmodon hispidus*), water rats (*Hydromys chrysogaster*), and spiny rats (*Proechimys guyanensis*) [56]. However, whether these species serve as true reservoirs or are merely incidental hosts during outbreaks remains unclear. It is possible that other rodent species, or even other as yet unidentified hosts in nature, could serve as natural reservoirs for the virus (Fig 4).

Overall, current evidence indicates that rodents play a central role in the ecology and dissemination of EMCV, particularly as sources of environmental contamination that facilitate spillover into susceptible hosts. However, their role as stable reservoirs in natural ecosystems, as well as the mechanisms underlying viral persistence between outbreaks, remains incompletely understood and warrant further investigation.

### 4.5. Other potential reservoirs

In addition to rodents, certain bat species, such as *Miniopterus fuliginosus,* have also been suspected of being potential reservoir hosts or carriers of EMCV in the wild (Fig 4) [58].

However, unlike the relatively robust body of research on rodents, available data on EMCV in bats remain limited and inconclusive.

EMCV has also been found in arthropods, particularly mites and mosquitoes [10,42], including various wild animal species such as wild boar (*Sus scrofa*) [50,59]. Nevertheless, experimental investigations in mosquitoes have provided no evidence of viral transmission. The presence of the virus in these arthropods is most plausibly explained by the ingestion of blood meals from infected hosts [10].

## 5. Pathogenicity of EMCV

EMCV infection in susceptible hosts is characterized by acute, often severe disease with high mortality, primarily associated with cardiac and, less frequently [1,15,16,51], neurological involvement [10]. In NHPs, the most commonly reported outcome is sudden death, frequently occurring without preceding clinical signs, particularly in zoological and captive settings [7,13,17–19].

When clinical signs are observed, they are typically nonspecific and rapidly progressive, including lethargy, anorexia, marked weakness, and respiratory distress consistent with cardiac dysfunction [14,15,17,47]. Reproductive disorders, including fetal loss, have been reported in certain species such as baboons [17,47].

Postmortem examinations consistently reveal severe myocarditis, which represents the principal lesion associated with EMCV infection in NHPs. Additional pathological findings may involve multiple organs, including the lungs, liver, spleen, kidneys, skeletal muscles, and, in some cases, the central nervous system [12,14,47,48,61]. These findings highlight the multisystemic nature of the infection, although cardiac lesions remain the dominant and most clinically relevant feature.

Experimental studies further support the high virulence and broad host susceptibility of EMCV strains. Strains originally isolated from NHPs, such as EMCV Ruckert and the Mengo virus, have been shown to induce severe disease, including paralysis, myocarditis, and death in laboratory animals such as mice [9,10].

Similarly, EMCV strains derived from macaques have caused high mortality in multiple experimental models, including rodents and rabbits [17]. Conversely, strains originating from other species, such as pigs, have also demonstrated the capacity to induce severe disease in primates [46].

In summary, EMCV exhibits a high degree of virulence across susceptible species, with a consistent tropism for cardiac tissue. In NHPs, infection is typically characterized by a peracute to acute clinical course, high case fatality, and prominent myocardial involvement, making EMCV a significant cause of sudden death in captive NHPs populations.

## 6. Zoonotic potential and human exposure to EMCV

Current evidence suggests that EMCV has zoonotic potential, although its impact on human health appears to be limited and incompletely understood. To date, no confirmed cases of severe EMCV-associated myocarditis or acute fatal infections comparable to those observed in NHPs and other animal species have been conclusively documented in humans.

Nevertheless, serological studies indicate that human exposure to EMCV occurs across diverse geographical regions. For example, seroprevalence rates range from 6% in coastal areas to over 17% in rainforest regions have been reported in Peru [23]. In China, a large-scale study using ELISA detected anti-EMCV antibodies in approximately 30.56% of healthy individuals, with higher prevalence observed in older age groups [25]. Elevated seroprevalence has also been reported among specific occupational groups, including hunters in Austria (~15%) [28,30] and swine veterinarians in Mexico (~47%) [24].

Epidemiological data indicate that human exposure is primarily associated with environmental, certain socioeconomic indicators, such as housing construction materials and neighborhood characteristics that may facilitate rodent intrusion and professionnal activity [23,38–42], rather than confirmed direct transmission from infected animals. Identified risk groups include pig farmers, swine veterinarians, zoo personnel, hunters, and individuals living in close proximity to intensive animal production or areas with high rodent activity [11,23,28–30,60].

The zoonotic potential of EMCV is further supported by genetic evidence, with close similarity observed between viral strains isolated from humans and those from animal hosts. Notably, EMCV strains obtained from febrile patients in Peru have shown high genetic relatedness to strains identified in animal species, including European porcine isolates and those from bonobos in the Democratic Republic of the Congo [14,26]. In some instances, virus isolation, RNA detection or the presence of specific antibodies have been detected in individuals with direct contact with infected animals, including NHPs [7,11].

Despite this evidence of exposure, most reported human infections appear to be asymptomatic or associated with mild, nonspecific clinical manifestations, such as fever, chills, headache, nausea, and vomiting [11,26]. Overall, available data suggest that EMCV should be considered a potential zoonotic virus with low apparent pathogenicity in humans, where exposure is likely driven by environmental contamination, particularly through contact with rodent reservoirs or infected animal environments, rather than sustained direct animal-to-human transmission. However, important gaps remain regarding the frequency of infection, transmission pathways, and clinical significance in humans.

## 7. Laboratory diagnosis of EMCV

Several tests are employed to detect and confirm EMCV infection, targeting different types of clinical specimens in human and NHPs [16–19].

### 7.1. Molecular testing

EMCV RNA is routinely detected and characterized by RT-PCR and sequencing [4,16,42,51]. However, genomic data from human and NHPs remain scarce, with most sequences originating from older studies and only a few strains fully characterized. Partial and complete genomes, as well as specific gene segments, have been obtained from clinical samples in lemurs, bonobos, orangutans, macaques, baboons, and chimpanzees [4,14,16]. The majority of nucleotide sequence data currently available comes from studies in swine and rodents (Table 2) [4,62–65].

**Table 2:** Nucleotide sequence references of genomes and genes from EMCV strains isolated from NHPs, wild and domestic animals, humans, and arthropods.

Isolates/virus designation	N°acc. genome/gene	Geographical origin	Species	Year of isolation/collection/Submission
EMCV Ruckert	M81861.1	USA	Chimpanzee	1944/1992
EMCV ATCC VR-129B	KM269482.1	USA	Chimpanzee	1944/2014
Mengo M	L22089.1	Uganda	Macaque	1948/1993
EMCV Sing-M105-02	KC310738.1	Singapore	Orangutan	2002/2013
EMCV Sing-M100-02	KC310737.1	Singapore	Orangutan	2002/2013
EMCV SPU 64/03	GU181317.1	DRC	Bonobo	2003/2009
Mengo 3761IMP	KX231802.1	Russia	Baboon	2007/2016
EMCV 64771	KY201174	Italy	Macaque	2012/2016
EMCV ITA-041/13-VR	OL840527.1	Italy	Lemur	2013/2021
EMCV ITA-016/15-VR	OL840508.1	Italy	Lemur	2015/2015
EMCV Peru-2004/10855	EU979546.1	Peru	Human	2004/2008
EMCV Peru-2004/10854	EU979543.1	Peru	Human	2004/2008
EMCV KNP 17/94	OR924201.1	South Africa	African elephant	1994/2023
EMCV FRA/SIG/1/2013	MT250508.1	France	African elephant	2013/2020
EMCV FJ13	KF293299.1	China	Tiger	2013/2013
EMCV-C15	KU664327	China	Dog	2015/2016
EMCV KEL367	PP766467	Madagascar	Beats	2019/2024
EMCV AR3595/61	OQ858575.1	South Africa	Acarina	2023
EMCV-B variant	M22457.1	USA	Swine	1980/1989
EMC-D variant	M22458	USA	Swine	1980/1989
EMCV-30	AY296731	USA	Swine	1987/2003
EMCV pv21	X74312.1	Germany	Swine	1993/1993
EMCV BEL-2887A/91	AF356822	Belgium	Swine	2001
EMCV JZ1202	KF836387	China	Swine	2012/2013
EMCV-CBNU	DQ517424.1	Korea	Swine	2006/2006
EMCV GS01	KJ524643.1	China	Pig	2013/2014
EMCV 80843	KY201173	Italy	Porcupine	2012/2016
EMCV ITL-001/92	AJ006863.1	Italy	Red squirrel	1992/1998
EMCV PV2	X87335	Germany	Mouse	1985/1995
EMCV pEC9	DQ288856.1	USA	Mice	1995/2005
Mengo Rz pMwt	DQ294633.1	USA	Mice	2005
EMCV RD 1338	JX257003.1	Germany	Soft wood	2005/2012
EMCV 1086C	DQ835185	Belgium	Rat	2008
EMCV ZM12/14	LC585221.1	Zambia	Mouse	2012/2020
EMCV FRA/SIG/1/2014	MT250509.1	France	Rat	2013/2020
EMCV AN7402/61	OQ858577.1	South Africa	Mouse	2023

### 7.2. Cell culture and isolation

The first strain of EMCV, designated EMCV Ruckert (EMCV-R), was isolated in 1944 in Florida from a chimpanzee that died suddenly from acute myocarditis [9]. In 1946, a second strain referred to as Mengo virus, classified under the EMCV-1 serotype, was isolated from the cerebrospinal fluid of a paralyzed rhesus macaque at the Mengo Research Institute in Entebbe, Uganda [10]. Since these initial discoveries, EMCV strains have been progressively isolated from a wide variety of species worldwide, notably from both domestic [2,4,25,66] and wild animals [7,8,13,54,55], including small rodents [21,56] and even in humans [11,26]. In NHPs, virus isolation has been documented not only from chimpanzees [9], and rhesus macaques [10,15,17,48], but also from bonobos [14], orangutans [16], grivet [17], lemurs [18], barbary macaques [18,19], and baboons [17,47]. Virus isolation is typically performed using baby hamster kidney fibroblast (BHK-21) cells, African green monkey kidney (Vero) cell lines, and laboratory mice (Table 2). A range of clinical specimens has been used, including pleural fluid and spleen [9], cerebrospinal fluid [10], serum, whole blood, pericardial fluid, cardiac tissue [14], and tissue samples from the heart, lungs, brain, and liver [13,16,19,41].

In humans, EMCV has been successfully isolated from serum and blood samples of patients presenting with febrile illnes and neurological symptoms [11,26]. Most EMCV strains have been isolated from rats and pigs [4,21,62–64,67,68].

### 7.3. Immunohistochemical and serological tests

Serological and immunohistochemical methods have been employed to detect EMCV-specific antibodies or antigens in humans and NHPs species. The serological assays include immunofluorescence [13,16], hemagglutination inhibition [12,17], and virus neutralization [13,16,17].

In a study by Yeo et al [16], neutralizing antibody titers against the EMCV Sing-M105-02 isolate, originally isolated from orangutans, were detected in 22 of 122 zoo animals tested, including capybaras, chimpanzees, and orangutans. Similarly, neutralizing antibodies were observed in eight of 23 rhesus macaques, one captive grivet, and 19 baboons among 630 animals tested in Uganda and the USA [10,47]. In addition to serology, immunohistochemical testing has revealed the presence of EMCV antigens in tissue samples from infected NHPs [14].

### 7.4. Microscopic testing

Histopathological analyses have consistently demonstrated cardiac lesions, most notably myocardial involvement characterized by extensive, focal, or diffuse myocarditis [14–19].

These inflammatory changes in the myocardium are among the most consistent and indicative findings associated with EMCV infections. Furthermore, electron microscopy has enabled the visualization of virus particles within affected tissues of NHPs [14].

## 8. Treatment and prevention of EMCV

To the authors’ knowledge, there is currently no specific antiviral treatment or commercially available vaccine EMCV infection. Clinical management, as reported in the limited number of documented cases NHPs, remains largely supportive and symptomatic.

Given the potential impact of EMCV on captive and semi-captive NHPs populations particularly in zoological, settings considerable efforts have been made to develop preventive strategies. In this context, several vaccine candidates, including inactivated formulations and live attenuated strains, have been evaluated during natural outbreaks as well as under experimental conditions [7,69–72]. However, despite these efforts, none have yet achieved sufficient evidence of safety, efficacy, and scalability to support widespread implementation.

### 8.1. Treatment

Due to the rapid progression of EMCV infection, therapeutic intervention is often challenging, if not futile. In addition, the disease remains poorly recognized, and appropriate diagnostic tools are typically implemented only at advanced stages of infection or not at all in live animals resulting in significant delays in clinical management [56]. In the absence of specific antiviral therapy, treatment is primarily supportive and may be attempted in animals presenting with mild to moderate clinical signs. However, in most cases, euthanasia should be seriously considered, given the poor prognosis, particularly in individuals with cardiac involvement or heart failure [15,40,56]. Various therapeutic approaches have been described in the literature, although with limited success. For instance, intravenous administration of ceftriaxone (50 mg/kg) was attempted in two bonobos at the Lola ya Bonobo Sanctuary (Democratic Republic of the Congo) during the advanced stages of an acute respiratory syndrome characterized by tachypnea and dyspnea; both animals died within 24 hours of the onset of clinical signs [14]. In other reports, captive elephants and a capybara exhibiting compatible clinical signs succumbed to the disease within a few days despite receiving supportive treatment, including antibiotics, analgesics, and antispasmodics [6,73]. Overall, these observations highlight the limited efficacy of currently available therapeutic interventions and underscore the critical importance of early detection and preventive measures.

### 8.2. Vaccination Strategies Against EMCV

Efforts to develop vaccines against EMCV have been ongoing for several decades, with overall heterogeneous and often inconsistent outcomes. Various vaccine strategies have been explored, including autogenous inactivated vaccines derived from outbreak-specific viral isolates, as well as live attenuated formulations [7,69–72].

The first vaccination trials were initiated following an outbreak in a zoological setting in New Orleans, USA, involving multiple species, including NHPs, elephants, and lions [7]. Since then, several vaccine candidates have demonstrated the ability to induce humoral immune responses across a range of species, including NHPs.

However, despite these encouraging findings, evidence supporting long-term immunity and cross-protection against genetically diverse EMCV strains remains limited and inconsistent [13,56]. These limitations highlight the ongoing challenges in developing broadly effective and durable vaccination strategies against EMCV.

#### 8.2.1. Inactivated Vaccines.

A formalin-inactivated vaccine derived from an American strain of EMCV (ATCC-VR-124) was administered to several primate species, including brown lemurs (*Eulemur fulvus*), ruffed lemurs (*Varecia variegata*), black-handed spider monkeys (*Ateles geoffroyi*), black-and-white colobus monkey, and tufted capuchins (*Cebus apella*) [7]. A heterogeneous immune response was observed, with spider monkeys and lemurs developing moderate antibody titers (1:20–1:80) following booster vaccination. In contrast, some individuals, including a Sumatran orangutan, exhibited markedly higher titers (1:640). However, no detectable serological response was observed in other primate species, underscoring marked interspecific variability in vaccine immunogenicity [7].

In a separate study, an inactivated vaccine prepared using β-propiolactone from an Australian EMCV strain was evaluated in ungulates, marsupials, and chimpanzees. While immune responses varied across species, chimpanzees developed high and sustained neutralizing antibody titers persisting for up to 12 months post-vaccination, without any reported adverse effects [71].

Other vaccine formulations, including oil-emulsion preparations derived from elephant-origin EMCV isolates and inactivated with β-propiolactone, have been shown to induce prolonged humoral responses in rhesus macaques [69]. However, the absence of viral challenge studies significantly limits the interpretation of these findings in terms of true protective efficacy, and robust long-term follow-up data remain scarce. In addition, certain formulations, particularly those based on porcine strains and inactivated using binary ethyleneimine failed to elicit any detectable antibody response in macaques, highlighting the critical influence of both viral strain antigenicity and inactivation methodology on vaccine immunogenicity [69].

Finally, commercially available porcine vaccines previously used in the United States demonstrated encouraging immunogenicity in NHPs, particularly hamadryas baboons, with good tolerability and antibody persistence for up to six months [74].

#### 8.2.2. Live Attenuated Vaccines.

Live attenuated vaccine strategies against EMCV have predominantly been developed through genetic manipulation of Mengo virus, a closely related picornavirus within the same genus. These approaches are largely based on attenuation achieved by targeted modification of the poly(C) tract, a genomic element recognized as a key determinant of viral virulence and pathogenicity [46].

Among these candidates, the Mengo virus-derived vMC24 vaccine, characterized by a shortened poly(C) sequence, has demonstrated strong immunogenicity and protective efficacy across multiple species, including macaques, baboons, and domestic pigs [56,70]. Notably, in baboons, the vaccine-induced significantly higher neutralizing antibody titers compared with those elicited by inactivated EMCV formulations [70]. A larger study evaluating a derivative of this vaccine (vMC0), which lacks the poly(C) sequence, also reported encouraging but heterogeneous results in zoo animals. High neutralizing antibody titers were observed in certain primate species; however, marked interspecies variability in immune responses was noted [72]. Seroconversion, defined as a ≥4-fold increase in antibody titers, was inconsistent and strongly species-dependent. It was detected only in selected individuals, including a dromedary camel, Baird’s tapir (*Tapirus bairdii*), Cape porcupine (*Hystrix africaeaustralis*), and gerenuks (*Litocranius walleri*), as well as in several primate species such as collared mangabeys (*Cercocebus torquatus*), Angolan colobus monkeys (*Colobus angolensis*), and Diana monkeys (*Cercopithecus diana*) [72].

In contrast, no seroconversion was observed in common marmosets, mantled guerezas, siamangs (*Hylobates syndactylus*), or vaccinated lemurs, further highlighting the pronounced species-dependent variability in vaccine-induced immune responses [72].

However, neither the vMC24 nor the vMC0 vaccine is commercially available. In practice, most zoological institutions often in response to mortality events within their collections have resorted to the production of autogenous inactivated vaccines derived from locally isolated viral strains [40].

Despite experimental progress with inactivated and live attenuated vaccines, no vaccine against EMCV has yet been commercialized on a large-scale or is currently available on the market, underscoring the need for preventive biosecurity measures [14,18].

The apparently low incidence of the disease in most settings despite sporadic outbreaks associated with high mortality in zoological collections likely constitutes a major constraint to vaccine development and commercialization. This perceived epidemiological rarity reduces industrial incentives and limits investment.

In addition, significant economic and logistical constraints further limit vaccine development, as the costs associated with research, regulatory evaluation, and large-scale production are generally not justified by an epidemiological burden perceived as sporadic and unpredictable. Moreover, the marked antigenic variability among EMCV strains represents an additional challenge, complicating the demonstration of broad, consistent, and reproducible vaccine efficacy across divergent viral lineages.

Consequently, preventive strategies based on strict adherence to biosecurity protocols remain the cornerstone for preventing both the introduction and dissemination of the virus in animal habitats. These measures primarily aim to limit rodent access and reduce contamination of water and feed sources, particularly under captive and semi-captive conditions in NHPs [14,18,47].

## 9. Conclusions

This review compiles and analyzes the current body of knowledge on EMCV in NHPs. Available data indicate that EMCV represents a significant zoonotic pathogen for NHPs, as evidenced by multiple fatal outbreaks in both captive and semi-captive populations. This poses a serious conservation concern, especially for endangered primate species.

Although human infections are generally asymptomatic or mild, serological evidence suggests widespread exposure and potential zoonotic risk. The high pathogenicity of EMCV in NHPs, particularly its association with sudden death and acute myocarditis, highlights the need to include EMCV in the differential diagnosis of unexplained mortality events in these species.

In the absence of specific treatment or licensed vaccines, prevention remains the cornerstone of control, relying on rodent management, biosecurity reinforcement, and improved surveillance within a One Health approach.

Significant gaps persist in our knowledge, particularly regarding the ecological reservoirs, transmission dynamics at the wildlife-human interface, and environmental drivers of spillover. Future research should prioritize longitudinal surveillance in wild and captive primates, standardized diagnostic tools, and improved sero-epidemiological studies to better define exposure patterns. Furthermore, experimental studies on host susceptibility and pathogenesis across primate species are needed, alongside exploratory work on vaccine development and immune correlates of protection.

**Key points**

- Encephalomyocarditis virus (EMCV) is a zoonotic virus with a worldwide distribution but is largely overlooked, particularly in humans and non-human primates (NHPs).
- EMCV can cause severe clinical manifestations and high mortality in NHPs, representing an underestimated threat to both NHPs species and public health.
- Data on EMCV in human and NHPs are scarce and fragmented; the virus is rarely considered in the diagnosis of human and NHPs diseases, which limits the assessment of its epidemiological impact.
- This review provides a near-exhaustive synthesis of published data on EMCV in primates and highlights the need for increased attention to this virus, because of its ability to cause severe disease in NHPs and its potential risk to humans

**Key learning points**

- Encephalomyocarditis virus (EMCV), a *Cardiovirus* primarily transmitted by rodents, represents an emerging yet largely overlooked threat to non-human primates (NHPs).
- Multiple fatal outbreaks of high mortality EMCV infection have been reported in captive and semi-captive NHPs, where infection is frequently associated with acute myocarditis and sudden death.
- Although documented human cases remain rare, serological evidence suggests frequent human exposure to EMCV, highlighting its underestimated zoonotic potential.
- The absence of licensed vaccines or specific treatments makes prevention through rodent control and strengthened biosecurity measures essential.
- Major knowledge gaps persist regarding the natural reservoirs, transmission dynamics, and epidemiology of EMCV, underscoring the need for enhanced surveillance and further research.

**Top five papers**

1. Wells SK, Gutter AE, Soike KF, Baskin GB. Encephalomyocarditis virus: epizootic in a zoological collection. *J Zool Wild Med*. 1989;20:291–296.
2. Reddacliff L, Kirkland PD, Hartley WJ, Reece R.L. Encephalomyocarditis virus infections in an Australian zoo. *J Zoo Wild Med*. 1997;28:153–157.
3. Jones P, Cordonnier N, Mahamba C, Burt F.J, Rakotovao F, Swanepoel R, André C, Dauger S, Bakkali Kassimi L. Encephalomyocarditis virus mortality in semi-wild bonobos (*Pan paniscus*). *J Med Primatol*. 2011;40:157–163.
4. Canelli E, Luppi A, Lavazza A, Lelli D, Sozzi E, Martin AM, et al. Encephalomyocarditis virus infection in an Italian zoo. *Virol J*. 2010;7:64.
5. Oberste MS, Gotuzzo E, Blair P, Nix WA, Ksiazek TG, Comer JA, et al. Human febrile illness caused by encephalomyocarditis virus infection, Peru. *Emerg Infect Say*. 2009;15(4):640-646.
